# Outcomes and factors leading to graft failure in kidney transplants from deceased donors with acute kidney injury—A retrospective cohort study

**DOI:** 10.1371/journal.pone.0254115

**Published:** 2021-08-26

**Authors:** Cheol Woong Jung, Dana Jorgensen, Puneet Sood, Rajil Mehta, Michele Molinari, Sundaram Hariharan, Armando Ganoza, Dirk Van Der Windt, Martin N. Wijkstrom, Chethan M. Puttarajappa, Amit D. Tevar

**Affiliations:** 1 Department of Surgery, Korea University Anam Hospital, Seoul, Korea; 2 Department of Surgery, Thomas E Starzl Transplantation Institute, University Pittsburgh Medical Center, Pittsburgh, Pennsylvania, United States of America; 3 Department of Internal Medicine, Thomas E Starzl Transplantation Institute, University Pittsburgh Medical Center, Pittsburgh, Pennsylvania, United States of America; University of Toledo, UNITED STATES

## Abstract

Due to shortage of donor, kidney transplants (KTs) from donors with acute kidney injury (AKI) are expanding. Although previous studies comparing clinical outcomes between AKI and non-AKI donors in KTs have shown comparable results, data on high-volume analysis of KTs outcomes with AKI donors are limited. This study aimed to analyze the selection trends of AKI donors and investigate the impact of AKI on graft failure using the United states cohort data. We analyzed a total 52,757 KTs collected in the Scientific Registry of Transplant Recipient (SRTR) from 2010 to 2015. The sample included 4,962 (9.4%) cases of KTs with AKI donors (creatinine ≥ 2 mg/dL). Clinical characteristics of AKI and non-AKI donors were analyzed and outcomes of both groups were compared. We also analyzed risk factors for graft failure in AKI donor KTs. Although the incidence of delayed graft function was higher in recipients of AKI donors compared to non-AKI donors, graft and patient survival were not significantly different between the two groups. We found donor hypertension, cold ischemic time, the proportion of African American donors, and high KDPI were risk factors for graft failure in AKI donor KTs. KTs from deceased donor with AKI showed comparable outcomes. Thus, donors with AKI need to be considered more actively to expand donor pool. Caution is still needed when donors have additional risk factors of graft failure.

## Introduction

Kidney transplantation is the most effective treatment to improve the survival and quality of life for patients with end-stage renal disease [1–3]. However, the discrepancy between donor demand and supply is causing an increase in waiting time for kidney transplantation [4]. As a result of long waiting time, many candidates become ineligible for due to deteriorating clinical status [5]. To address this problem, there is increased interest for the use of kidneys from marginal donors including donors with AKI [6–8]. Previous studies have shown that clinical outcomes are comparable between AKI and non-AKI donors in KTs [9–11]. Nevertheless, nearly one-fifth of kidneys recovered with intent to transplant are not used, totaling 3159 discarded kidneys in 2015, despite evidence that some of these discarded kidneys could benefit wait-listed patients [12, 13]. There are limited data on high-volume analysis of KTs outcomes with AKI donor. In addition, there is a lack of accurate methods to assess kidney quality. These might explain some of the high-discard rates in AKI donor. To support better clinical decision making and prevent unnecessary discard, more evidence is needed. The objective of this study was to compare clinical outcomes in KTs from deceased donors with or without AKI and to identify risk factors for graft failure in AKI donor KTs. Results of this study will provide information to improve clinical decision making and to clarify whether AKI kidneys should be used.

## Materials and methods

### Data set

This study used data obtained by the SRTR for kidney transplant recipients from 2010 to 2015. The SRTR data system includes data on all donors, waitlisted candidates, and transplant recipients in the US submitted by members of OPTN and has been previously described in detail [14]. Health and Resources Services Administration, US Department of Health and Human Services provides oversight to activities of OPTN and SRTR contractors.

### Study population

A total of 87,272 adult recipients who underwent KT between January 1st, 2010 and December 31st, 2015 were evaluated for inclusion in this study. We excluded living donors (n = 29,620), those with missing donor creatinine (n = 643), incompatible blood groups (n = 5), and those with implausible values (n = 4,247). A total of 52,757 recipients were included in the main analysis. The sample was further divided into two groups based on donor serum creatinine at allocation: 1) Non-AKI donor serum creatinine < 2 mg/dL; and 2) AKI donor serum creatinine ≥ 2 mg/dL.

### Statistical methods

Clinical and demographic characteristics of the two groups are presented as frequencies and proportions (categorical variables), as mean and standard deviation or 95% confidence interval [95%CI] for normally distributed variables, or median and interquartile range [IQR] for skewed distributions. χ^2^, Kruskal-Wallis rank-sum, and Student’s t-tests were used to compare group differences in recipient, donor, and transplant characteristics. Due to large number of patients in AKI and non-AKI groups, a more conservative p-value of *p* < 0.0001 were considered significant. For comparisons within the AKI group among those with and without graft failure, *p* < 0.05 was considered significant. Kidney Donor Profile Index (KDPI) was calculated based on OPTN guidelines [15]. The 2015 reference population for the KDPI and scaling factor were used for converting KDRI rao to KDRI median (1.2175005163). Kaplan-Meier survival analyses were performed to compare patient and graft survival between the two groups. Log rank tests with p-values < 0.05 were considered significant. Survival estimates were calculated for 1, 2, 3, and 4-year periods.

Logistic regression with backward selection was used to model the odds of graft loss among the AKI group. We entered significant predictors from Table 3 into the model, including donor gender, donor age, mechanism of death (CVA, anoxia, other), glomerular sclerosis, donor creatinine, donor hypertension status, cold ischemia time (hours), donor AA race, recipient AA race, and recipient age. *P*-values < 0.05 were considered significant predictors of graft failure. Additionally, we evaluated KDPI both continuously and as a categorical variable (KDPI>85 vs ≤84) in separate models. For models including KDPI, variables included in the calculation of KDPI were excluded to avoid collinearity. All analyses for this paper were completed using SAS 9.4 (SAS Institute Inc., Cary, NC, USA).

## Results

This study cohort consisted of 52,757 adult deceased donor kidney transplants (DDKTs) in the U.S. alone between January 1, 2010 and December 31, 2015. Four thousand nine hundred sixty-two (9.4%) KTs were performed using donors with AKI. The control group included 47,795 non-AKI donor KTs.

### Comparison of baseline characteristics between AKI and non-AKI groups

The median age of the AKI group was significantly lower than that of the non-AKI group, 35.7 years [95%CI: 35.4, 36.1] vs 40.3 [95%CI: 40.1, 40.4] years respectively, *p* < 0.0001. In addition, proportions of extended criteria donors (ECDs) and those with hypertension (HTN) history over 10 years were lower in the AKI group compared to those in the non-AKI group (12.3% vs. 16.4%, *p* < 0.0001 and 14.1% vs. 19.1%, *p* < 0.0001, respectively) (Table 1). Fewer recipients with a high PRA (PRA > 50%) or a history of prior KT were observed in the AKI group compared to the non-AKI group (17.1% vs. 22.3%, *P* < 0.0001 and 10.2% vs. 13.2%, *P* < 0.0001, respectively) (Table 1). This may be reflective of the fact that high PRA recipients have a greater chance to be matched with a better kidney. More biopsies were performed in the AKI group (92.3% vs. 46.7%) to identify the causes of high creatinine. AKI donors were more likely to have high KDPI (48.0% vs. 44.0%, *P* < 0.0001) and roughly 34% of AKI kidneys were from non-local donors. Consequently, AKI group had a higher odds of pump use (51.8% vs. 40.6%, *P* < 0.0001). Finally, cold ischemic time (CIT) was longer in AKI group (18.9 [IQR: 13.3, 26.0] hours vs. 15.3 [IQR: 10.4, 21.2] hours, *p* < 0.0001) (Table 1).

**Table 1:** Donor, recipient and transplant characteristics.

	non-AKI	AKI*	p-value
	N = 47795	N = 4962	
Donor characteristics			
Age (years), mean (95% CI)	40.3 (40.1, 40.4)	35.7 (35.4, 36.1)	<0.0001
Male (%)	59.2	71.0	<0.0001
Race (%)			<0.0001
White	69.4	61.6	
African American	13.9	20.0	
Hispanic	13.3	14.9	
Asian	2.5	2.2	
Other	1.0	1.3	
BMI (kg/m^2^), mean (95% CI)	27.9 (27.8, 27.9)	29.4 (29.2, 29.6)	
Expanded Criteria donors (%)	16.4	12.3	<0.0001
KDPI (%), median [IQR]	44.0 [22.0, 69.0]	48.0 [31.0, 69.0]	<0.0001
Diabetes (y/n) (%)	7.9	7.3	
Diabetes>10 years (%)	1.65	1.39	
Hypertension (y/n) (%)	29.5	27.9	
Hypertension > 10yrs (%)	19.1	14.9	<0.0001
Cause of Death (%)			<0.0001
Anoxia	27.45	47.42	
Cerebrovascular	33.70	21.89	
Head Trauma	35.80	28.13	
Proteinuria (%)	43.6	62.4	<0.0001
Hepatitis C virus (%)	2.8	0.9	<0.0001
Creatinine (mg/dL), mean (95% CI)	0.96 (0.95, 0.96)	3.25 (3.20, 3.30)	<0.0001
Recipient characteristics			
Age (years), mean (95% CI)	53.9 (53.8, 54.1)	54.4 (54.0, 54.7)	
Male (%)	61.2	61.5	
Race (%)			<0.0001
White	44.2	39.2	
African American	32.4	33.7	
Asian	6.3	6.9	
Hispanic	15.3	18.1	
Other	1.8	2.1	
BMI (kg/m^2^), mean (95% CI)	28.3 (28.2, 28.4)	28.4 (28.3, 28.6)	
Diabetes (%)	36.5	37.3	
Hypertension (%)	72.2	75.1	
Time on waitlist (months), median [IQR]	27.0 [10.0, 45.0]	27.0 [11.0, 46.0]	
High PRA > 50%	22.3	17.1	<0.0001
Prior KT (%)	13.2	10.2	<0.0001
Transplant characteristics			
HLA mismatch, median [IQR]	4.0 [3.0, 5.0]	4.0 [4.0, 5.0]	
HLA mismatch >3 (%)	72.5	75.9	<0.0001
KDPI (%), median [IQR]	44.0 [22.0, 69.0]	48.0 [31.0, 69.0]	<0.0001
Glomerular sclerosis (%)			NA
Missing data (%)	53.3	7.7	
≤10	37.7	72.4	
10–20	6.4	7.4	
≥20	2.6	2.6	
Pump right kidney (%)	35.8	48.0	<0.0001
Pump left kidney (%)	35.5	47.7	<0.0001
Organ share type (%)			
Local	78.9	66.4	<0.0001
Regional	8.8	15.5	<0.0001
National	12.3	18.2	<0.0001
CIT (hours), median [IQR]	15.3 [10.4, 21.2]	18.9 [13.3, 26.0]	<0.0001
CIT>20 hours (%)	28.5	43.9	<0.0001

Abbreviation: AKI, acute kidney injury; CI, confidence interval; BMI, body mass index; KDPI, kidney donor profile index; IQR, interquartile range; PRA, panel reactive antibody; HLA, human leukocyte antigen; NA, not applicable; CIT, cold ischemic time

*AKI was defined as serum creatinine level of ≥2.0 mg/dL

### Comparison of clinical outcomes between AKI and non-AKI groups

The incidence of delayed graft function (DGF) was significantly higher in the AKI group (44.6% vs. 24.5%, *p* < 0.0001). However, acute rejection rate, BKV, and CMV infection rate were similar between the two groups (all *p’s* >0.0001). Although creatinine at the time of discharge was higher in the AKI group compared to that in the non-AKI group (4.9 ± 3.0 mg/dL vs. 3.3 ± 2.8 mg/dL, *p* < 0.0001), the last follow-up creatinine was not significantly different between the two groups (Table 2).

**Table 2:** Transplant outcomes.

	non-AKI	AKI*	p-value
	N = 47795	N = 4962	
DGF (%)	24.5	44.6	<0.0001
Acute Rejection (%)	18.0	16.5	
BKV infection, (n, %)	3430 (7.2%)	340 (6.9%)	
CMV infection, (n, %)	7981 (16.7%)	887 (17.9%)	
Creatinine at discharge (mg/dL), mean ± std	3.3 ± 2.8	4.9 ± 3.0	<0.0001
Creatinine Decline≥25% in First 24 hours (%)	31.8	19.6	<0.0001
Follow-up Creatinine (mg/dL), mean ± std	1.68 ± 0.91	1.64 ± 0.87	
Follow-up Creatinine (mg/dL), median [IQR]	1.49 [1.20, 1.90]	1.41 [1.16, 1.84]	

Abbreviation: AKI, acute kidney injury; DGF, delayed graft function; BKV, BK virus; CMV, cytomegalovirus

*AKI was defined as serum creatinine level of ≥2.0 mg/dL

### Comparison of death-censored graft survival and patient survival between AKI and non-AKI groups

**Fig 1:**
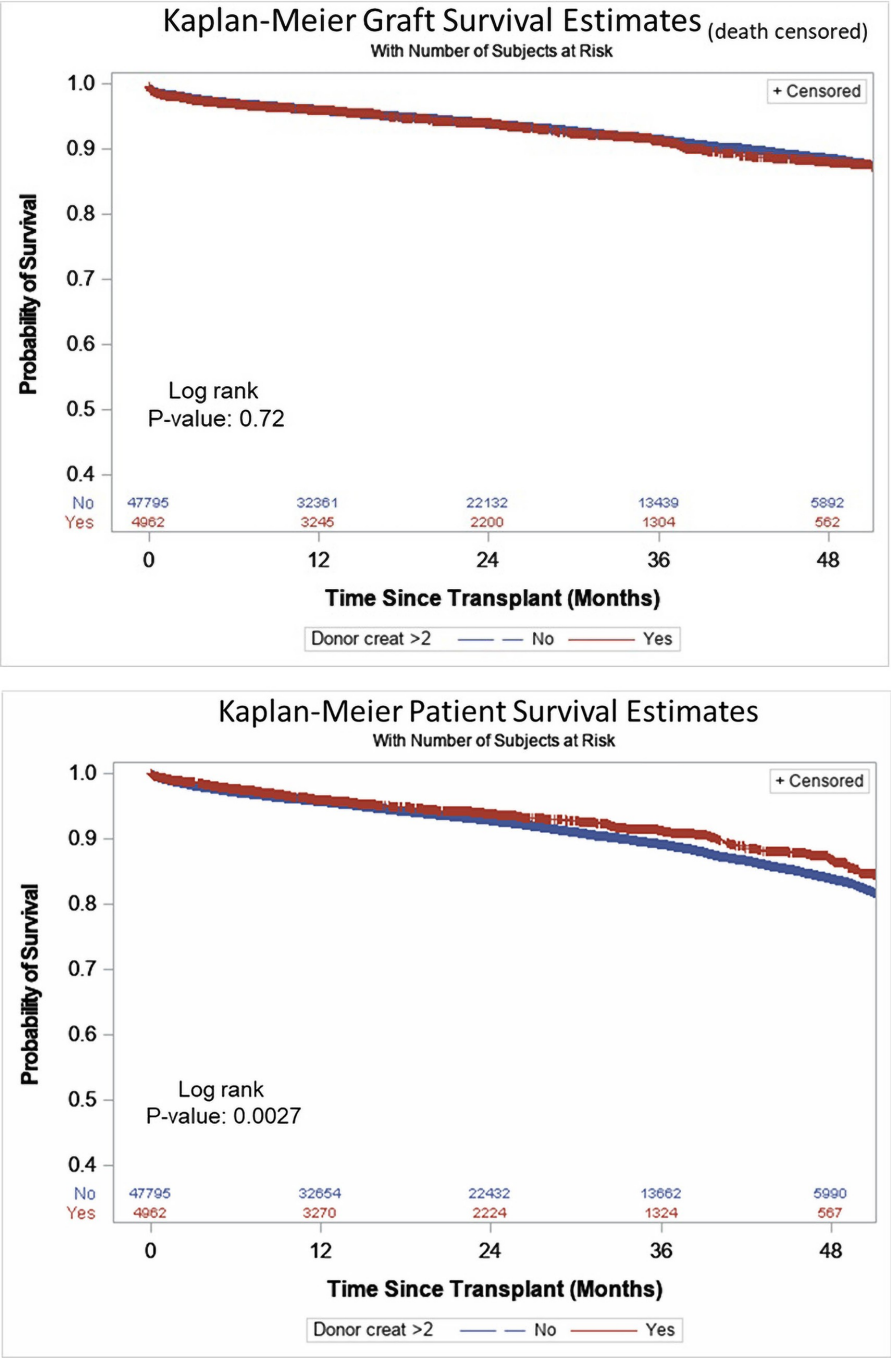
Kaplan–Meier curves of death-censored graft survival and patient survival compared for donors with and without AKI. A. Death-censored graft survival. B. Patient survival.

Kaplan-Meier estimates of death-censored graft survival and patient survival between two groups were then determined. There was no statistically significant difference between the two groups. All groups showed greater than 80% graft and patient survival during the follow-up period. Median follow up period of this study was 24.4 (IQR: 12.2 to 36.6) months (Fig 1).

### Risk factors predicting graft failure among AKI donors

Compared to the no graft failure group, donors in the graft failure group were significantly older, 37.67 [95%CI: 36.18, 39.16] vs 35.58 [95%CI: 35.21, 35.95] years respectively, p < 0.0049. The proportion of donors aged more than 60 years was also higher in the graft failure group (3.6% vs. 2.2%, *p* < 0.02). The proportion of African-American (AA) donors and ECDs was also higher in the graft failure group (30.4% vs. 19.3%, *p* < 0.0001 and 17.6% vs. 11.9%, *p* < 0.002, respectively). KDPI and HTN rate were both higher in the graft failure group (57.0 [37.0, 80.0] vs. 47.0 [31.0, 67.0], *p* < 0.0001 and 39.5% vs. 27.0%, *p* < 0.0001, respectively). The proportion of brain death from cardiovascular event was higher and mean CIT was longer in the graft failure group (29.8% vs 21.3%, *p* < 0.0035 and 20.1 [14.6, 27.1] vs. 18.8 [13.2, 26.0] hours, *p* < 0.01) (Table 3). Acute rejection rate was higher in the graft failure group (45.15% vs.14.04%, *p* < 0.0001) (Table 3).

**Table 3:** Donor, recipient and transplant risk factors predicting graft failure in AKI donor KTs.

	Non graft fail	Graft failed	p-value
	N = 4633	N = 329	
Donor risk factors			
Age (years), mean (95% CI)	35.58 (35.21, 35.95)	37.67 (36.18, 39.16)	0.0049
Age > 60 (n, %)	102 (2.20%)	17 (3.59%)	0.02
Male (n, %)	3304 (71.31%)	220 (66.87%)	0.09
Race (n, %)			< .0001
White	2886 (62.29%)	169 (51.37%)	
African American	894 (19.30%)	100 (30.40%)	
Asian	100 (2.16%)	7 (2.13%)	
Hispanic	691 (14.91%)	49 (14.89%)	
Other	62 (1.34%)	4 (1.22%)	
BMI (kg/m^2^), mean ± std	29.36 ± 6.99	29.57 ± 7.15	0.60
Extended Criteria donors (n, %)	551 (11.89%)	58 (17.63%)	0.0022
KDPI (%)	47.00 [31.00, 67.00]	57.00 [37.00, 80.00]	< .0001
Diabetes (n, %)	1738 (37.5%)	113 (34.4%)	0.25
Hypertension (n, %)	1252 (27.02%)	130 (39.51%)	< .0001
Proteinuria (n, %)	2889 (62.36%)	205 (62.31%)	1.00
Glomerular sclerosis (n, %)			0.03
Missing data	837 (18.07%)	39 (11.85%)	
≤10	3341 (72.11%)	251 (76.29%)	
10–20	338 (7.30%)	27 (8.21%)	
≥20	117 (2.53%)	12 (3.65)	
Cause of Death (n, %)			
Cerebrovascular	988 (21.33%)	98 (29.79%)	0.0035
Hepatitis C virus (n, %)	41 (0.88%)	4 (1.22%)	0.54
CIT (hours), median [IQR]	18.80 [13.23, 26.00]	20.06 [14.55, 27.11]	0.01
CIT >20hrs (n, %)	2018 (43.94%)	163 (50.31)	0.03
Machine perfusion (n, %)	2390 (51.59%)	180 (54.71%)	0.27
Organ Share type (n, %)			
Local	3054 (66.32%)	220 (67.28%)	0.72
Regional	713 (15.48%)	49 (14.98%)	0.81
National	838 (18.20%)	58 (17.74)	0.83
Creatinine(mg/dL), mean ± std	3.27 ± 1.90	2.96 ± 1.57	0.0008
Creatinine(mg/dL), median [IQR]	2.60 [2.20, 3.60]	2.50 [2.20, 3.10]	0.0261
Recipient risk factors			
Age (years), mean (95% CI)	54.50 (54.13, 54.86)	52.83 (51.37, 54.30)	0.02
Male (n, %)	2842 (61.34%)	210 (63.83%)	0.37
Race (n, %)			0.0038
White	1806 (38.98%)	140 (42.55%)	
African American	1544 (33.33%)	129 (39.21%)	
Asian	331 (7.14%)	11 (3.34%)	
Hispanic	852 (18.39%)	44 (13.37%)	
Other	100 (2.16%)	5 (1.52%)	
BMI (kg/m^2^), mean ± std	28.40 ± 5.14	28.86 ±5.20	0.60
Prior KTs (n, %)	471 (10.17%)	37 (11.25%)	0.53
Diabetes (n, %)	1738 (37.51%)	113 (34.35%)	0.25
Hypertension (n, %)	3480 (88.96%)	246 (89.13%)	0.92
Time on waiting list (months), median [IQR]	27.00 [11.00, 46.00]	27.00 [10.00, 44.00]	0.40
PRA > 50% (n, %)	216 (4.66%)	18 (5.47%)	0.0006
Transplant risk factors			
HLA mismatch, median [IQR]	4.00 [4.00, 5.00]	5.00 [4.00, 5.00]	0.48
HLA mismatch >3, (n, %)	4959 (89.17%)	439 (92.62%)	0.06
Acute Rejection, (n, %)	781 (14.04%)	214 (45.15%)	< .0001

Abbreviation: AKI, acute kidney injury; CI, confidence interval; BMI, body mass index; KDPI, kidney donor profile index; CIT, cold ischemic time; IQR, interquartile range; KT, kidney transplant; PRA, panel reactive antibody; HLA, human leukocyte antigen

Logistic regression was performed to identify potential risk factors for graft failure among the AKI group while adjusting for confounding factors. We found that donor creatinine, donor HTN, CIT, AA donor, and acute rejection were significant predictors for graft failure in the AKI group (all p’s<0.05). A higher donor creatinine was associated with lower odds of graft failure (OR:0.91; 95%CI:0.83,0.99). While donor HTN, CIT and AA donor were all associated with a higher odds of graft failure (Table 4). In a separate model, we examined KDPI as a predictor for graft failure. In the model examining KDPI as a continuous variable, we observed that for every increase in the KDPI by 1, the odds of graft failure increase by 1% as well. In the model examining KDPI categorically (over 85% compared to less than or equal to 85%), the odds of graft failure are doubled in those with a KDPI over 85% (OR:2.07, 95%CI:1.30, 3.30) (Table 4).

**Table 4:** Multivariable logistic regression models predicting the odds of graft failure in the AKI donor KTs. A. Including all significant predictors from Table 3 except KDPI. B. Adding KDPI and removing the variables included in the calculation of KDPI to avoid collinearity. C. Adding KDPI (>85%) and removing the variables included in the calculation of KDPI to avoid collinearity.

A.					
Factors	Odds ratio	95% Wald			Pr > ChiSq
		Confidence Limits			
Donor creatinine	0.91	0.83		0.99	0.04
Donor hypertension	1.46	1.12		1.89	0.005
Cold ischemia time	1.01	1.00		1.02	0.04
African American donor	1.67	1.27		2.20	0.0003
Acute rejection (Any)	4.93	3.74		6.51	< .0001
B.					
Factors	Odds ratio	95% Wald			Pr > ChiSq
		Confidence Limits			
KDPI (for every 1% increase)	1.01	1.00	1.02		0.0002
African American recipient	1.57	1.10	2.24		0.01
Acute rejection (Any)	4.18	2.80	6.26		0.01
C.					
Factors	Odds ratio	95% Wald			Pr > ChiSq
		Confidence Limits			
KDPI > 85%	2.07	1.30	3.30		0.0021
African American recipient	1.57	1.10	2.24		0.0134
Acute rejection (Any)	4.36	2.92	6.52		< .0001

## Discussion

In the era of donor shortage, efforts to expand the donor pool has continued. Increasing awareness in the potential use of kidneys from marginal donors, including those with AKI, has been increased accordingly [6, 7, 16]. Nevertheless, the discard rate of donor kidneys with elevated creatinine levels remains high. According to SRTR annual report, the discard rate of kidneys recovered for transplant with high creatinine level (creatinine >1.5 mg/dL) is around 34%. In contrast, such rate is only around 14% for kidneys with low creatinine level (creatinine ≤1.5 mg/dL) [4]. A previous analysis of UNOS data by Kayler et al., found that among donors with a terminal creatinine >2.0 mg/dL neither kidney was recovered 44% of the time. This is in comparison to only 2.1% and 4.9% among donors with creatinine levels ≤1.5 mg/dL and 1.6–2.0 mg/dL, respectively. Kayler et al. mentioned that donor terminal creatinine level was a significant independent predictor of kidney discard despite adjusting for other confounding factors [17]. Using the SRTR data, we found that donor kidneys with favorable factors such as young age, non-ECD, and shorter CIT were more common in transplantation of donors with high creatinine level. Therefore, it is conceivable that AKI kidneys with more favorable parameters are selected for KTs.

Lower graft survival in the AKI group has been previously found in a study of 1,869 kidney with AKI in the UK [8]. Graft survival was only 2% lower than that in the non-AKI group. Authors concluded that such reduced graft survival could be accepted considering an annual death rate of 8.2% in those remaining on the transplant waiting list [8]. Recently, Hall et al. published a multicenter cohort study after analyzing outcomes of 585 deceased-donor kidneys with AKI. They concluded that the current practice of using donor AKI kidneys was not associated with reduced allograft survival. In addition, pre-specified variables did not modify the effect of donor AKI on graft survival [11]. Consistent with previously published literatures, our results also showed that death-censored graft survival or patient survival in AKI donors was not inferior to that of non-AKI donors, although DGF rates were high. Despite the findings, the discard rate of AKI kidneys has not been significantly changed [13]. It might be due to the general conception that AKI is a predisposing factor of chronic kidney disease (CKD) in combination with other factors that may compound the impact of AKI [7, 18, 19].

Our study intends to provide clinicians ample information to assist in deciding whether to use AKI kidneys. This data is based on a registration generalizable to the US population. In logistic regression analysis, risk factors for graft failure in AKI donors were AA donors and donors with HTN, CIT, and acute rejection episode. Among risk factors for graft failure in the AKI group, donor factors included AA race, HTN, and CIT. When we examined KDPI in separate models: AA recipient, KDPI, and acute rejection were found to be risk factors for graft failure in AKI donors.

The incidence of ESRD in AA is four times higher than in whites [20]. Previous studies have shown inferior graft and patient outcomes associated with a transplant of AA donor kidneys [20–22]. Callender et al. have reported that five-year graft survival rates for Blacks AA were only 60.9% with a disparity of as much as 13–19% compared to other ethnic groups [21]. Similarly, a recent study using SRTR data indicated that AA donor race was associated with increased all-cause mortality and cardiovascular mortality and graft loss [22]. The most acceptable explanation on the inferior outcome of AA donors in KTs is an unfavorable genetic background of AA donors such as apolipoprotein L1 gene (APOL1) and non-muscle myosin IIA gene (MYH9) variants [23–25]. Besides, AAs suffer from more comorbidities than whites such as HTN, diabetes, and sickle cell trait, resulting in more ischemic events [26].

Long CIT was one risk factor for predicting graft failure. In an analysis of SRTR data from 2005 to 2015, Dube et al. found there was an increased risk of graft failure when the CIT exceeded 36 hours. It was predictive of death-censored graft survival (DCGS) (aHR: 1.27, *P* = 0.03) in multivariate analysis [27]. The analysis from a French observational multicenter prospective cohort noted that there was a significant proportional increase in the risk of graft failure for each additional hour of CIT (hazard ratio: 1.013) [28]. However, another study using SRTR data showed that DCGS between short and long CIT group was not significantly different regardless of the extent of CIT difference [29]. In our study, CIT was a statistically significant risk factor for graft failure (odds ratio: 1.011, 95% CI:).

After analyzing SRTR data, Rao et al. have proposed kidney donor risk index (KDRI) for deceased donor kidneys to quantify graft failure risk based on donor and transplant characteristics [30]. Since KDPI was adapted for kidney allocation in December 2014, it has been used to determine whether to accept or discard a kidney. However, KDPI still has only moderate predictive power (c = 0.60). It lacks the ability to differentiate kidneys of similar KDPI values with high confidence [31]. Massie et al. have shown that recipients with the highest KDPI kidneys have lower overall cumulative mortality than equivalent patients who forego high-KDPI KT in hope of receiving a lower KDPI kidney [32]. Analyzing UNOS data, Jay et al. have reported that preKT and non-preKT KDPI > 85% transplant are associated with lower mortality hazard after the first year compared with the waitlist including KDPI 0–85% transplants in patients >60 years old [33]. Therefore, KDPI itself should not be the only reason to discard a kidney, as long as, kidneys with a high KDPI provides a survival benefit over remaining on the waitlist for certain groups of patients.

This study has potential limitations. First, registry data were used. Therefore, we are limited to exploring only the factors collected and there may be selection bias. Selection bias might have occurred from the moment of donor allocation for AKI donor according to the selection policy of each institution. Such favourable graft outcomes and survival in the AKI group in this study may, in part, reflect a careful selection of AKI kidney. One of results possibly driven from this selection bias was that the mean creatinine level was statistically higher in the non-graft fail group. We attempted to address confounding by adjusting for other donor factors using statistical techniques. We found some relations with creatinine and CIT with the scatter plot. It showed that high creatinine donor kidneys were correlated with lower CIT. Besides, there might be some other unmeasured confounders. Second, we could not adopt AKI definition or stage in this study because SRTR data provided only a single pretransplant creatinine level. Consequently, we defined AKI as a terminal creatinine greater than or equaled 2 mg/dL instead of using the recent AKI definition such as AKIN criteria. However, as shown in the biopsy rate (92.3% in the AKI group), we can expect that most donors with high creatinine caused by chronic kidney disease were excluded depending on the biopsy findings. Third, SRTR does not provide sequential follow-up creatinine values. Thus, we could not measure or compare graft function after KTs. Follow-up creatinine shown in Table 3 is the mean/median creatinine level measured in the blood drawn from the most recent patient visit. Although SRTR required follow-up creatinine to be entered into the database annually, if patient was lost during the follow-up, the last creatinine level would continue to be recorded.

Nevertheless, our study suggested that KTs from deceased donor with AKI showed comparable outcomes with good long-term allograft survival. Graft failure in AKI donor KT was higher in AA donor and donor with HTN and long CIT. Creatinine was not a weighty factor in predicting graft failure in the AKI group. Thus, the use from donors with AKI needs to be considered more actively to expand the donor pool. Caution is needed when donor is AA or has HTN, long CIT, and obviously high KDPI.
